# Introducing Artificial Intelligence into Surgical Practice for Cancer Care: Clinical Applications and Ethical Considerations

**DOI:** 10.3390/jcm15218396

**Published:** 2026-10-28

**Authors:** Harrison Kulinski, Bryan Lim, Warren M. Rozen

**Affiliations:** Department of Plastic and Reconstructive Surgery, Peninsula University Hospital Bayside Health, Frankston 3199, Australia

**Keywords:** LLMs, skin cancer, melanoma, SCC, BCC, ChatGPT

## Abstract

**Background/Objectives:** Large language models (LLMs) are increasingly used in healthcare, but their utility in plastic surgery and skin cancer management remains in contention. Furthermore, the comparative performance of LLMs in the setting of skin cancer surgery is unclear. This study assessed four widely used LLMs and comparatively evaluated their relevance, accuracy, and clinical representativeness. **Methods:** This prospective study evaluated six standardised skin cancer prompts submitted to ChatGPT, Gemini, Claude, and Copilot. Specialist plastic surgeons assessed textual responses for relevance and accuracy, and generated images for clinical representativeness and accuracy. Readability and DISCERN scores were also assessed. **Results:** All four LLMs produced highly relevant textual responses, with relevance scores ranging from 4 to 5, while accuracy scores ranged from 3 to 5. ChatGPT and Copilot achieved the highest individual accuracy scores. Only ChatGPT and Gemini generated images. ChatGPT demonstrated greater clinical representativeness and accuracy across all image prompts. ChatGPT produced the most linguistically complex responses, while Copilot produced the most readable outputs. All models generated text requiring at least a high-school level of education. Claude achieved the highest mean DISCERN score (43.67), followed by ChatGPT (40.33), Copilot (36.00), and Gemini (35.67). **Conclusions:** In this exploratory comparison, contemporary LLM-based platforms produced relevant and largely accurate skin cancer surgery information, but differed in image generation, readability, and information quality. The generated images depicted predominantly lighter skin tones, which warrants greater input from more diverse patient populations, and outputs should be used alongside clinician review. Larger studies using patient-specific scenarios and diverse skin tones are needed.

## 1. Introduction

Artificial intelligence (AI) has rapidly emerged as a transformative technology with the potential to reshape healthcare delivery and clinical practice. Within surgery, its applications span the entire patient journey, from outpatient assessment and diagnostic decision-making to preoperative planning, intraoperative assistance, postoperative monitoring, and long-term follow-up [1,2]. Advances in large language models (LLMs), computer vision, and machine learning have further expanded the potential applications of AI beyond conventional data analysis, allowing these systems to process complex clinical information, synthesise medical literature, and generate context-specific recommendations [3]. As surgical practice continues to evolve, AI may, therefore, represent not only a tool for improving existing workflows, but also a means of identifying opportunities for innovation across the surgical pathway.

Plastic surgery, with its diverse clinical scope and combination of diagnostic, technical, and reconstructive decision-making, represents a particularly relevant field for exploring these capabilities. AI has been investigated across multiple areas of plastic surgery, including reconstructive planning, flap selection, imaging interpretation, wound assessment, surgical education, outcome prediction, and patient communication [4,5,6]. In addition to supporting established clinical processes, generative AI may offer a distinct opportunity to assist surgeons in exploring emerging concepts, synthesising disparate areas of knowledge, and identifying potential gaps in current practice [4,5,6]. However, the clinical utility of AI-generated information depends on its ability to provide responses that are not only relevant and accurate, but also appropriately contextualised and sufficiently original to contribute meaningfully to surgical advancement [7].

Despite growing enthusiasm surrounding AI in surgery, important questions remain regarding whether these systems can move beyond reproducing established knowledge to generate genuinely innovative ideas. Generative models are trained predominantly on existing information and may, therefore, favour commonly described approaches, potentially resulting in convergent rather than divergent thinking [7,8]. Furthermore, the generation of plausible but inaccurate or unverifiable information, including erroneous references, represents an important limitation when considering their use in academic and clinical settings [7,8,9,10]. Previous studies have evaluated either the accuracy of LLM-generated text in response to isolated clinical questions or the recognisability of AI-generated dermatological images [11,12]. Less is known about how current LLM-based platforms compare when asked to describe, illustrate, and explain the surgical management of the three main skin malignancies, and about the readability and quality of the information they provide. This exploratory study, therefore, compared four widely used platforms (ChatGPT, Gemini, Claude, and Copilot) in describing the appearance and surgical treatment of basal cell carcinoma, cutaneous squamous cell carcinoma, and melanoma. We assessed the relevance and accuracy of text responses, the clinical representativeness and accuracy of generated images, and readability and information quality, with a view to informing the potential role of these tools in patient and medical education.

## 2. Materials and Methods

This prospective study evaluated the capabilities of multiple LLMs across a diverse range of plastic-surgery-related topics using a standardised set of six prompts. The prompts were designed to reflect clinically and academically relevant questions encountered within plastic surgery, with additional prompts specifically developed to assess the models’ ability to generate visual representations of skin cancers. Each prompt was submitted independently to each LLM using a standardised methodology, and the complete textual and image-based outputs were collected for subsequent evaluation. Given the substantial advances in generative AI and multimodal model capabilities over recent years, the image-generation component was included to evaluate the contemporary ability of LLMs to produce clinically representative dermatological and oncological imagery.

Four commercially available LLM platforms were selected for evaluation: ChatGPT (OpenAI, San Francisco, CA, USA), Gemini (Google, Mountain View, CA, USA), Claude (Anthropic, San Francisco, CA, USA), and Copilot (Microsoft, Redmond, WA, USA). These platforms were selected because they represent four of the most widely used and accessible generative AI systems and are among the leading commercially available LLM platforms at the time of the study [11]. Their inclusion also enabled comparison across major AI developers with established multimodal and image-generation capabilities. For consistency, the publicly accessible versions of each platform available during the study period were used, with the specific model/version and access modality documented at the time of data collection. All responses were generated during 28–29 August 2026, using ChatGPT version GPT-5.6 Luna, Gemini version 3.6 Flash, and Claude (Anthropic’s) version Sonnet 5 on Medium. Copilot (Version 19.2610.35011) has no listed version name, and its Copilot chat feature was utilised to generate all responses.

The textual responses generated by each LLM were independently assessed by specialist plastic surgeons with extensive clinical and research experience. Five-point Likert scales were used because they provide a structured and reproducible framework for specialist assessment of subjective constructs, including relevance, accuracy, clinical representativeness, and image accuracy, while allowing gradations in perceived performance to be distinguished. This framework was designed to determine whether LLMs function primarily as tools for synthesising existing knowledge or whether they may also contribute to the identification of new concepts and potential avenues for innovation within plastic surgery, and if the responses are suitable for patient and medical education.

The generated skin cancer images were independently reviewed by specialist plastic surgeons using predefined assessment criteria. Evaluators assessed the images using Likert scales designed to evaluate their clinical representativeness and accuracy in depicting the specified skin cancer pathology. This component was intended to explore the emerging visual-generation capabilities of contemporary image-generation models and their potential application in surgical education, clinical communication, and representation of cutaneous malignancies. The image-generation assessment was considered separately from the evaluation of textual responses.

Objective linguistic characteristics of the textual responses were assessed using established readability indices. The Flesch Reading Ease Score, Flesch–Kincaid Grade Level, and Coleman–Liau Index were calculated for each response to assess readability and the level of education required for comprehension. The DISCERN score was used as a criterion for assessing the suitability and reliability of the information provided. These objective measures were evaluated alongside specialist surgeon assessments to provide a multidimensional assessment of LLM performance.

Statistical analyses were performed using IBM SPSS Statistics for Windows, version 30. These analyses compared the performance of the different LLMs across the assessed domains. Inter-rater agreement between the two assessors was calculated using weighted Cohen’s kappa for each Likert domain (relevance, accuracy, and clinical representativeness). In the setting of a discrepancy by one point, a second round of assessment was performed to reach consensus.

## 3. Results

Six individual prompts were used across the four LLMs assessed: ChatGPT, Gemini, Claude (Anthropic), and Copilot (Table 1). All responses were generated during 28–29 August 2026, using ChatGPT version GPT-5.6 Luna, Gemini version 3.6 Flash, and Claude (Anthropic’s) version Sonnet 5 on Medium. Copilot has no listed version name, and its Copilot chat feature was utilised to generate all responses. Not all models generated text-based output for every prompt. For prompts eliciting purely graphical responses, textual readability and DISCERN metrics could not be calculated and were recorded as not applicable (N/A).

### 3.1. Likert Scale Scores

#### 3.1.1. Likert Scale Scores—Graphical Responses

Three of the six prompts (prompts 1, 3, and 5) focused on image generation and description of cSCC, cBCC, and melanoma. Only ChatGPT and Gemini generated images of the requested skin cancer variant (Figure 1, Figure 2, Figure 3, Figure 4, Figure 5 and Figure 6). Claude (Anthropic) and Copilot declined synthetic images, with both stating concerns for potentially misleading the public, or concerns of using for self-diagnosis of potentially harmful skin lesions.

Generated images were assessed for clinical representativeness and accuracy in depicting the specified pathology using a 5-point Likert scale (Table 2). ChatGPT outperformed Gemini across both criteria for every prompt: clinical representativeness for ChatGPT ranged from 3 to 4, compared with 1 to 3 for Gemini, and accuracy scores ranged from 3 to 5 for ChatGPT compared with a consistent score of 2 for Gemini. The lowest scores for Gemini were observed for SCC (prompt 3, clinical representativeness = 1), with Gemini uniformly scoring a low 2/5 in accuracy across all prompts, suggesting limited fidelity in depicting these pathologies compared with ChatGPT’s outputs.

#### 3.1.2. Likert Scale Scores—Textual Responses

The next 3 of the 6 prompts (2, 4, and 6) focused on generating textual responses detailing the gold standard surgical treatment for different types of skin cancers (Supplementary Figures S1.1–S4.6).

Across the three treatment-focused prompts, all four LLMs produced textual responses that were assessed for relevance and accuracy using a 5-point Likert scale (Table 3). Relevance scores were uniformly high, ranging from 4 to 5 across all models and prompts, indicating that each LLM reliably addressed the clinical question posed. Accuracy scores were similarly favourable, ranging from 3 to 5, with ChatGPT and Copilot achieving the highest individual scores (5/5) for prompt 6.

Inter-rater agreement varied across domains between the two expert raters for both generated images and generated textual responses. All agreements were exact or within 1 point (Table 4). However, chance-corrected agreement was moderate to high, with weighted Cohen’s kappa between 0.50 and 0.93. However, the wide confidence intervals reflect the limited number of prompts and small number of independent raters.

### 3.2. Readability Scores

Objective readability metrics were calculated for all textual responses, using the Flesch Reading Ease Score, Flesch–Kincaid Grade Level Score, and Colemon–Liau Index (Table 5, Table 6 and Table 7) [13].

On the Flesch Reading Ease Score, mean scores were lowest (i.e., most difficult to read) for ChatGPT (15.83, SD ± 4.88), whilst Copilot had the highest mean score (41.17, SD ± 5.76), which resulted in producing the most readable responses overall (Table 5). Mean grade levels were highest for ChatGPT (12.93, ±SD 0.81), with Copilot again producing the simplest text (9.43, SD ± 1.06).

However, it is important to mention that all LLMs produced significantly complex responses, requiring at least a high-school level of education, with ChatGPT requiring a tertiary level of education on average amongst the responses assessed.

The Coleman–Liau Index, which estimates readability based on character-per-word and sentence-length metrics, similarly identified ChatGPT as generating the most linguistically complex responses (mean 18.89, ±SD 1.32), and Copilot (16.57, SD ± 0.82; Table 7).

Looking at these three readability scores together, ChatGPT consistently generated the most linguistically complex textual responses when compared to the three other LLMs assessed, with Copilot generating the most readable textual responses across all validated scoring systems utilised.

### 3.3. DISCERN Scores

The DISCERN score is a 16-question tool used to assess the quality and reliability of written information relating to medical treatment decisions, with implications for subsequent patient and medical practitioner education [14]. As a result, this score was only applied to the prompts focusing on the description of skin cancer treatment (prompts 2, 4, and 6), with higher scores reflecting a higher-quality, more reliable source of information.

Claude (Anthropic) had the highest mean of 43.67 (SD ± 6.03), followed by ChatGPT (40.33, ±SD 5.03), Copilot (36.00, ±SD 6.08), and Gemini (35.67, ±SD 2.52; Table 8).

## 4. Discussion

The increasing integration of artificial intelligence into surgical practice has created significant interest in its capabilities to support clinical decision-making, patient education, and research innovation. Plastic surgery has enormous breadth, covering reconstructive, oncological, and aesthetic practice. This setting demands not only accurate synthesis of established evidence but also nuanced clinical judgement, individualised decision-making, and, at times, genuinely novel problem-solving.

This study evaluated the performance of four widely used LLMs across a series of plastic-surgery-related prompts, assessing relevance, accuracy, image-generation capability, readability, and information quality. Overall, the findings suggest that contemporary LLMs are highly capable of producing relevant and largely accurate clinical information but potentially show a consistent and notable limitation in generating images and textual responses that truly reflect the diverse patient base that plastic surgeons treat. Previous work has largely focused on the accuracy of LLM-generated responses to isolated clinical questions, or on the appearance of AI-generated dermatological images, with fewer studies examining text and image outputs together. Less is known about how contemporary LLM-based platforms compare in the setting of skin cancer surgery, including the readability and quality of the information they provide. This study addresses part of that gap through a small exploratory comparison of four widely used platforms.

### 4.1. Implications for Clinical Practice: Readability, Reliability, and Clinical Applicability

The readability findings demonstrated that the textual responses generated by LLMs largely required tertiary-level education to comprehend the text. These more linguistically challenging responses can potentially raise the baseline medical literacy of patients who consult them before or between clinical appointments, enabling more informed questions and improved shared decision-making [15]. Alternatively, if the patient has a lower level of formal education, this can create more confusion, making shared decision-making and informed consent more challenging [16]. This increased complexity was most apparent in the generated responses from ChatGPT.

Beyond readability, the generated responses were largely considered accurate, especially when discussing the surgical management of melanoma with an average score of 4.5/5 on a 5-point Likert scale (Table 3). Because the prompts were generic, individualisation could not be assessed, but none of the responses mentioned the anatomical exceptions to recommended margins that guidelines include. This was evident in the discussion of wide local excision margins for melanoma. All responses linked the recommended margin to the relevant histology, but none noted that in some anatomical locations, proximity to critical structures precludes excision of the full recommended margin (Supplementary Figures S3.6 and S4.6).

This has direct implications for clinical workflow. Patients may present with fixed expectations shaped by AI regarding how they believe their skin cancer should be managed. This can result in additional time spent explaining the clinical nuance and importance of individualised surgical planning, while also potentially requiring clinicians to re-establish patient trust and manage expectations where the proposed treatment differs from what the patient was led to expect.

The DISCERN scores reflect this sentiment. Claude (Anthropic) had the highest mean score (43.67, SD ± 6.03). However, with only three scored responses per model, the study was likely underpowered to detect meaningful differences; therefore, these scores should be interpreted as descriptive observations only.

### 4.2. Divergent Behaviour and Ethical Considerations of Synthetic Image Generation

The four models assessed during this study diverged greatly in their willingness to generate synthetic images of skin cancers. Claude (Anthropic) and Copilot refused to generate synthetic images, both citing concerns over patient safety and their potential to facilitate self-diagnosis and healthcare avoidance (Supplementary Figures S3.1, S3.3, S3.5, S4.1, S4.3, and S4.5), whilst ChatGPT and Gemini generated images relevant to the prompts assessed (Figure 1, Figure 2, Figure 3, Figure 4, Figure 5 and Figure 6).

Amongst the two models that generated synthetic images, the quality was deemed inconsistent. ChatGPT’s synthetic images were rated more favourably on a 5-point Likert scale across both clinical representativeness and accuracy (Table 2).

This difference in quality and accuracy is an important consideration, particularly in the setting of increasing patient utilisation of LLMs for their health information [13,17]. Increased patient use of AI models may improve accuracy and health literacy, but this benefit is contingent on image quality [17]. Poor-quality synthetic images risk increasing misdiagnosis and healthcare avoidance, as they may capture neither the full breadth of oncological phenotypes nor the clinical nuance required to manage them [18].

A further consideration is that these synthetic images do not represent real, biopsy-proven lesions; rather, they are generated outputs shaped entirely by the underlying model’s generative process. This means the accuracy of any given image is governed by processes that remain largely opaque, not only to patients and healthcare practitioners, but in important respects to the model developers themselves, who can rarely fully account for why a generative model produced one specific output rather than another [19,20,21]. This can potentially result in confusion between a benign and a potentially metastatic lesion. This risk is often underestimated and is also compounded by the increasing public perception that AI tools are inherently more accurate than human clinicians [22]. However, as demonstrated in the results of this study, the quality of the image generation can be variable, and no prompt demonstrated the required nuance necessary to inform patients about what management best suits them.

Where synthetic images currently have great potential use is in clinical education. By using AI-generated images in educational material, it bypasses the concerns of patient privacy and potentially allows for improved access to pseudo-clinical imagery without impacting patient confidentiality with appropriate expert curation, given that even the best-performing model did not achieve high levels of routine accuracy [23,24].

### 4.3. Apparent Limitations of LLMs in Skin Cancer Surgery

A limitation identified across all generated responses was that no AI model accounted for differences in skin tone or ethnicity. This was especially apparent amongst the synthetic images from ChatGPT and Gemini, with all generated images displaying white skin (Figure 1, Figure 2, Figure 3, Figure 4, Figure 5 and Figure 6). This is an important consideration, as whilst most skin cancers occur in Caucasian people, when people of colour develop skin cancers, they tend to have worse outcomes and have a poorer prognosis due to delayed diagnosis and poor public perception of who gets skin cancers [25]. Additionally, amongst patients of colour, skin cancers often present in sun-protected, less common sites, such as the volar aspect of the hand and forearm, sole of the feet, and nailbeds [26]. Only Claude (Anthropic) made mention of subungual melanoma, with no other model assessed mentioning these less common regions (Supplementary Figure S3.5). AI models that underrepresent differences in skin tone can be potentially harmful by disproportionately misrepresenting the appearance, prevalence, or clinical features of skin cancers across different racial and ethnic groups. Underrepresentation may reinforce existing social perceptions that skin cancer only affects lighter-skinned populations, while underrepresenting its occurrence in darker-skinned populations [12]. This may contribute to reduced awareness among clinicians and patients, potentially delaying recognition and diagnosis of skin malignancies in underrepresented populations. Furthermore, if AI-generated educational or clinical images predominantly depict skin cancers on lighter skin, these systems may perpetuate existing disparities in dermatological education and contribute to a cycle in which underrepresentation in training data leads to further underrepresentation in clinical practice [27].

### 4.4. Limitations

This study has several limitations. It is a small exploratory comparison and not a powered evaluation of LLM performance. Firstly, two plastic surgeons with extensive experience in skin cancer surgery evaluated the LLMs’ responses. Although their expertise maintains the study’s credibility, there may still be an element of subjectivity and bias present. This could have been combated through incorporating more raters, which would limit inter-rater variability. Inter-rater agreement was assessed on a small number of ratings, and most scores clustered around 4–5, which limits the interpretability of Cohen’s kappa scores. Secondly, each prompt was submitted once per platform, and only one image was generated per prompt. As outputs from LLM’s are stochastic, a single response or image does not characterise a platform’s range of outputs.

### 4.5. Directions for Future Research

The findings of this study, while informative, are drawn from a small sample of prompts and a single snapshot of four rapidly evolving platforms. This highlights the need for larger, more systematic research in this space. Future studies should evaluate a broader range of plastic surgery prompts across a wider range of pathologies, incorporate multiple independent raters with formal inter-rater reliability reporting, and track model performance longitudinally as platforms are updated, to determine whether the limitations identified here, inconsistent image quality, lack of representation across skin tones, and limited clinical nuance, are persistent structural features or ones that improve with future development. Additionally, whilst not the aim of this study, future research should design prompts aimed at asking the chosen LLM to identify research gaps or truly new ways of managing skin cancer to better analyse model novelty. Whilst this study has highlighted potential for LLM integration in skin cancer surgery, clinician, and patient education, until more evidence and greater consensus is made, LLMs are best positioned as a supervised adjunct to, rather than a replacement for, individualised clinical judgement and patient education.

## 5. Conclusions

This study highlights that LLMs produced relevant, largely accurate information on skin cancer surgery, but the four models differed in their readability and DISCERN scores. These differences may help guide future implementation of AI models in plastic surgery practice to aid in patient flow, medical, and patient education, which are all imperative to the field. It is essential to acknowledge that AI is not intended as a substitute for medical expertise; rather, its role is best realised alongside supervision in the setting of patient and medical education. Based on the current findings, there is no suggestion that changes to clinical care are currently warranted. Furthermore, as technology advances, LLMs may be utilised to generate novel solutions to the challenges faced by plastic surgeons in the management of skin cancer.

## Figures and Tables

**Figure 1:**
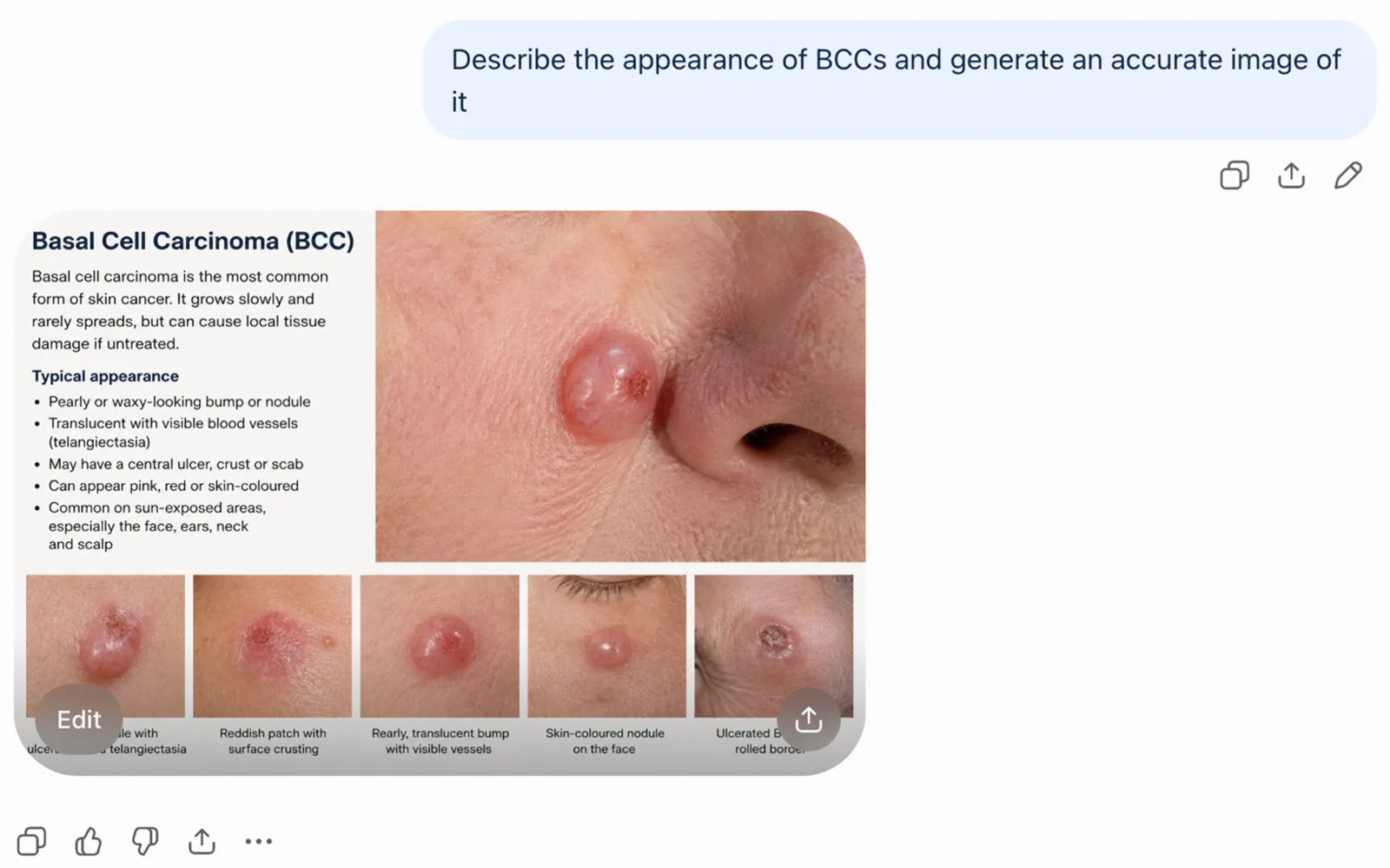
ChatGPT BCC images generated.

**Figure 2:**
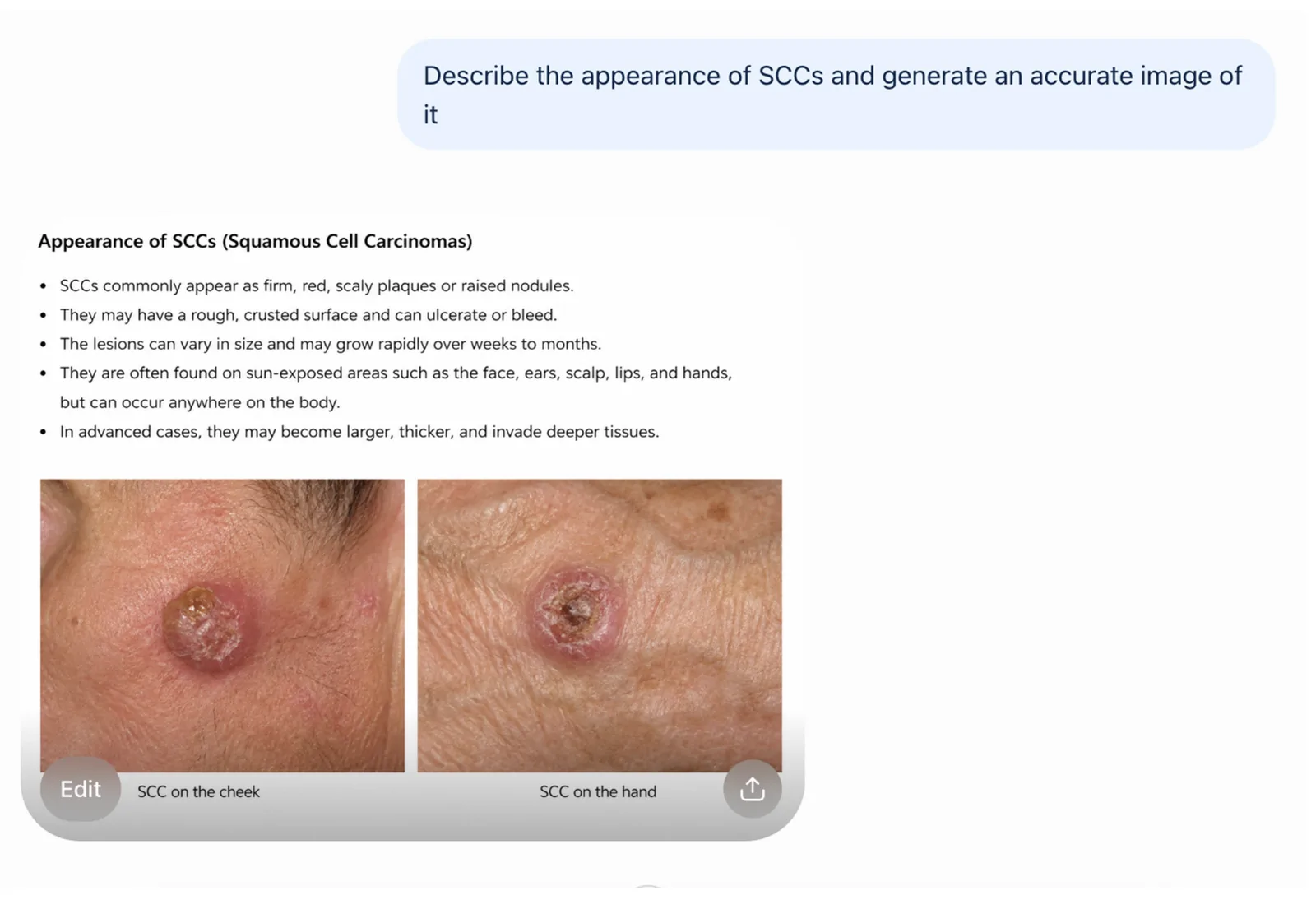
ChatGPT SCC images generated.

**Figure 3:**
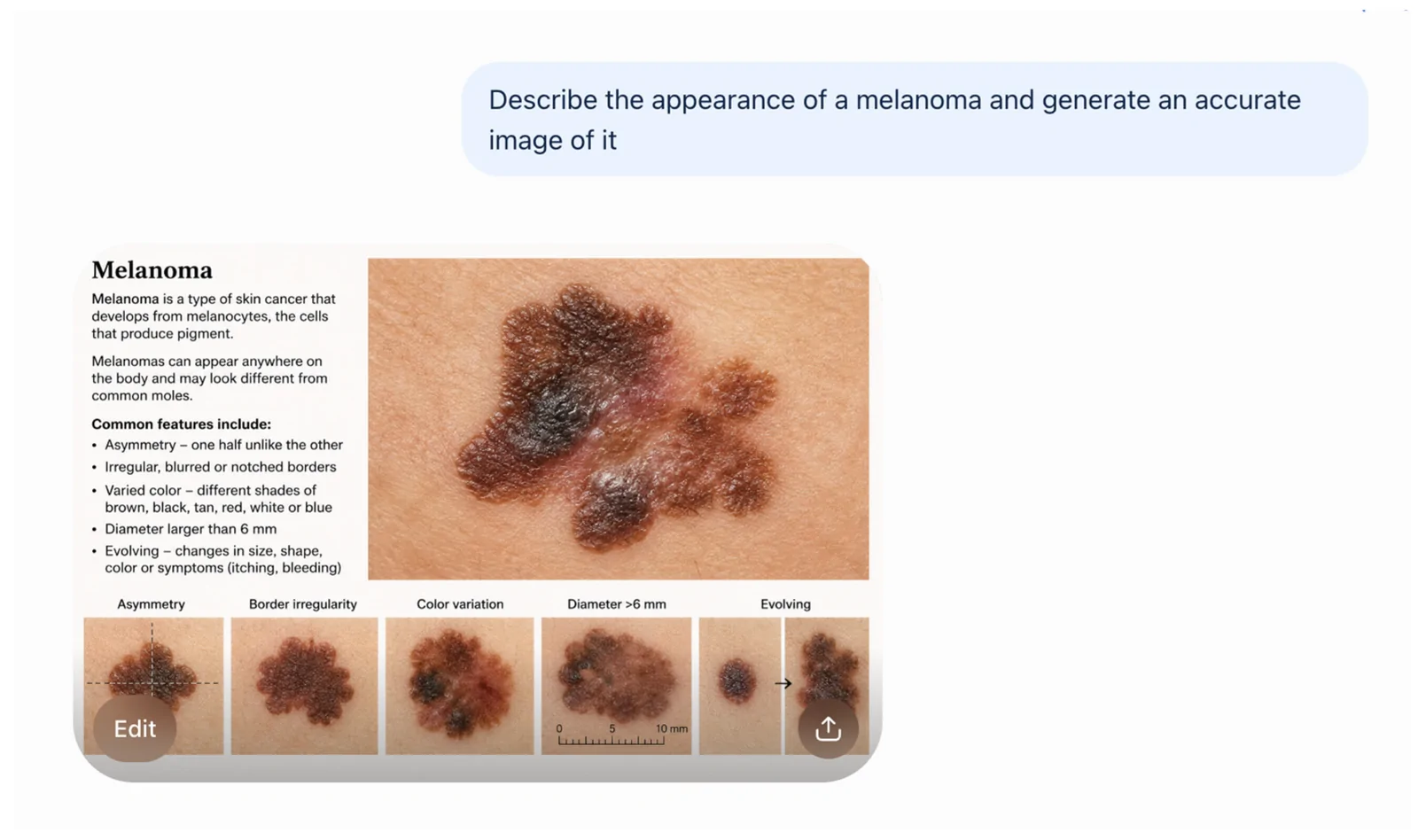
ChatGPT melanoma images generated.

**Figure 4:**
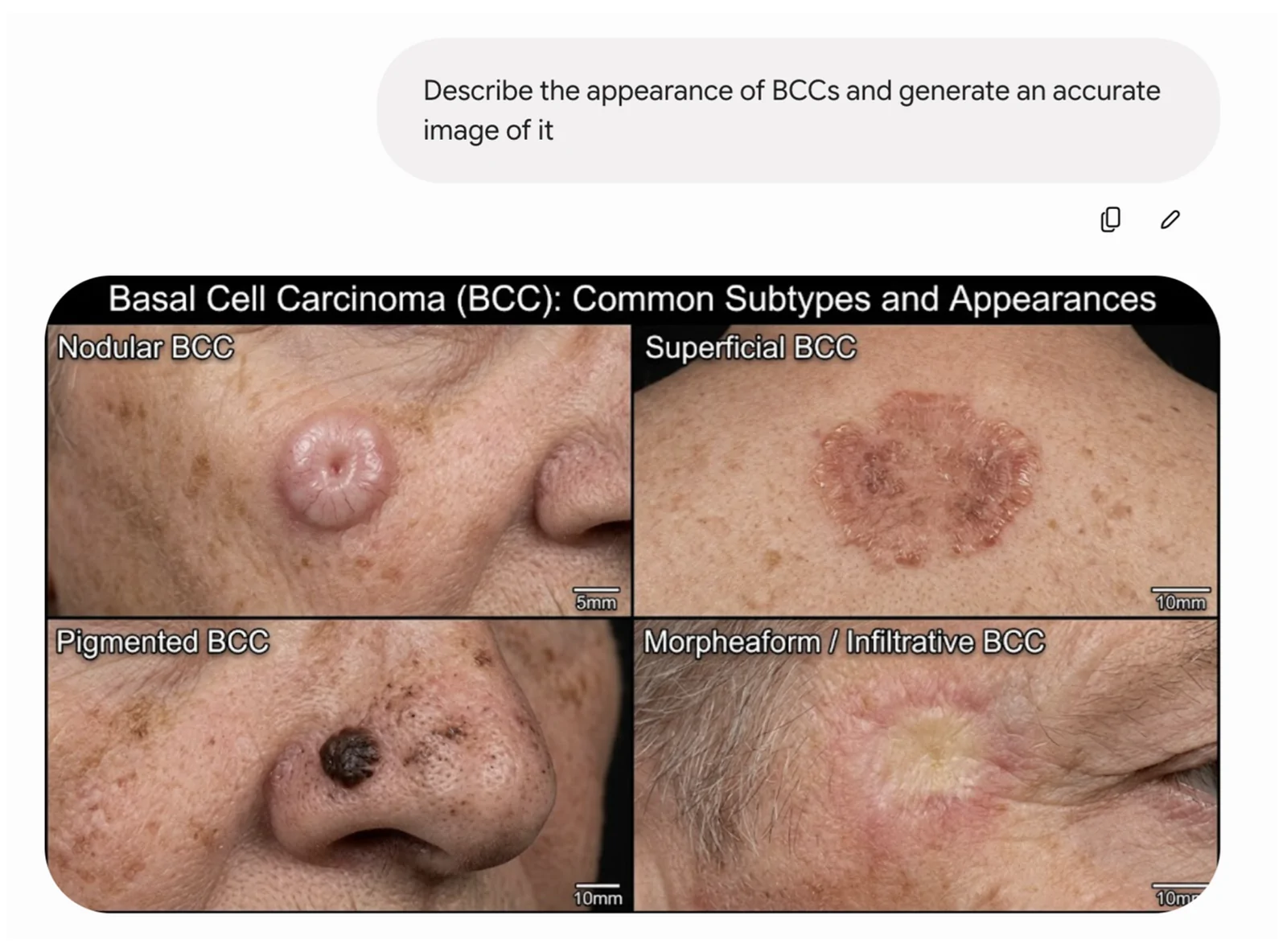
Gemini BCC images generated.

**Figure 5:**
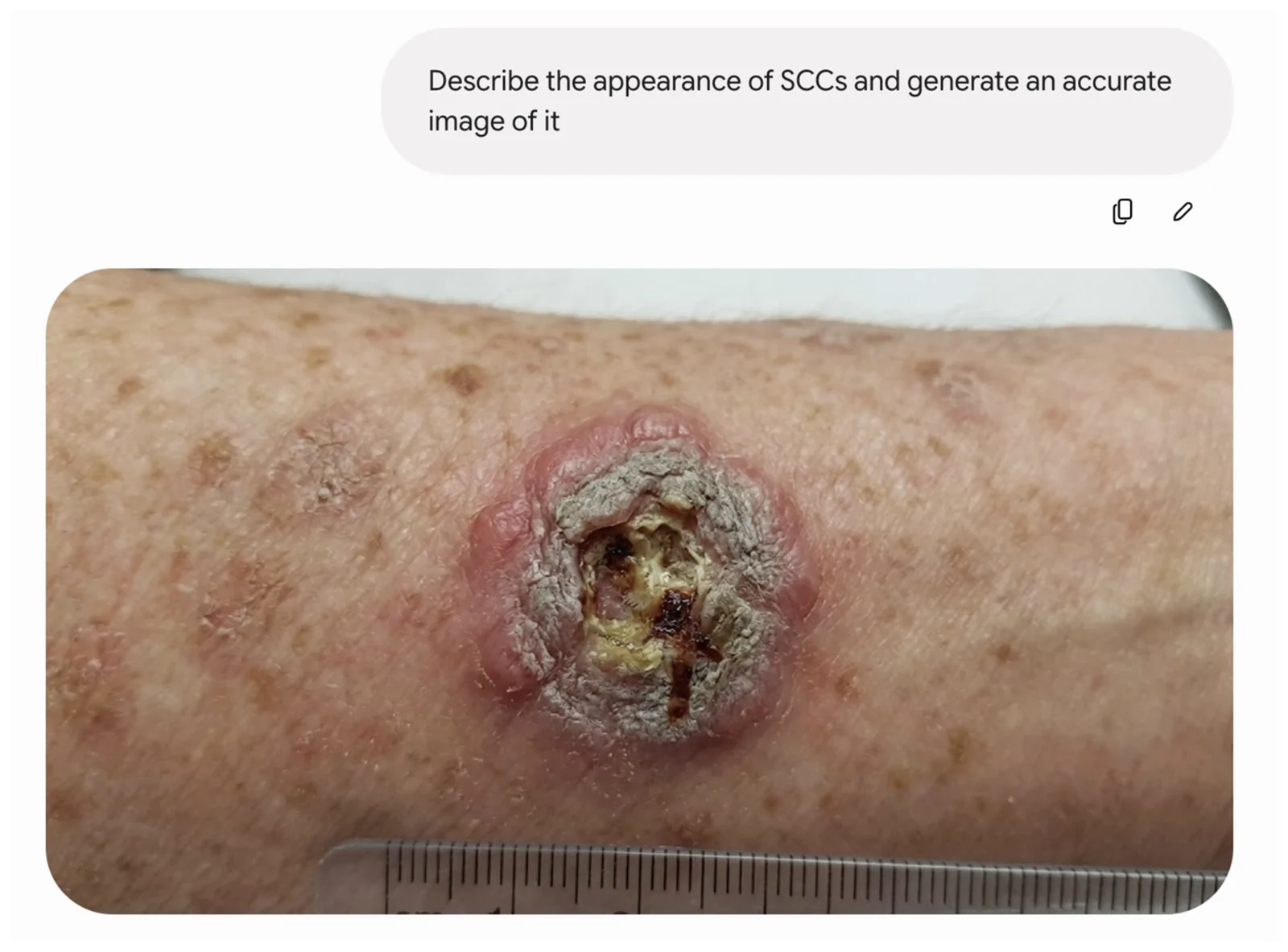
Gemini SCC images generated.

**Figure 6:**
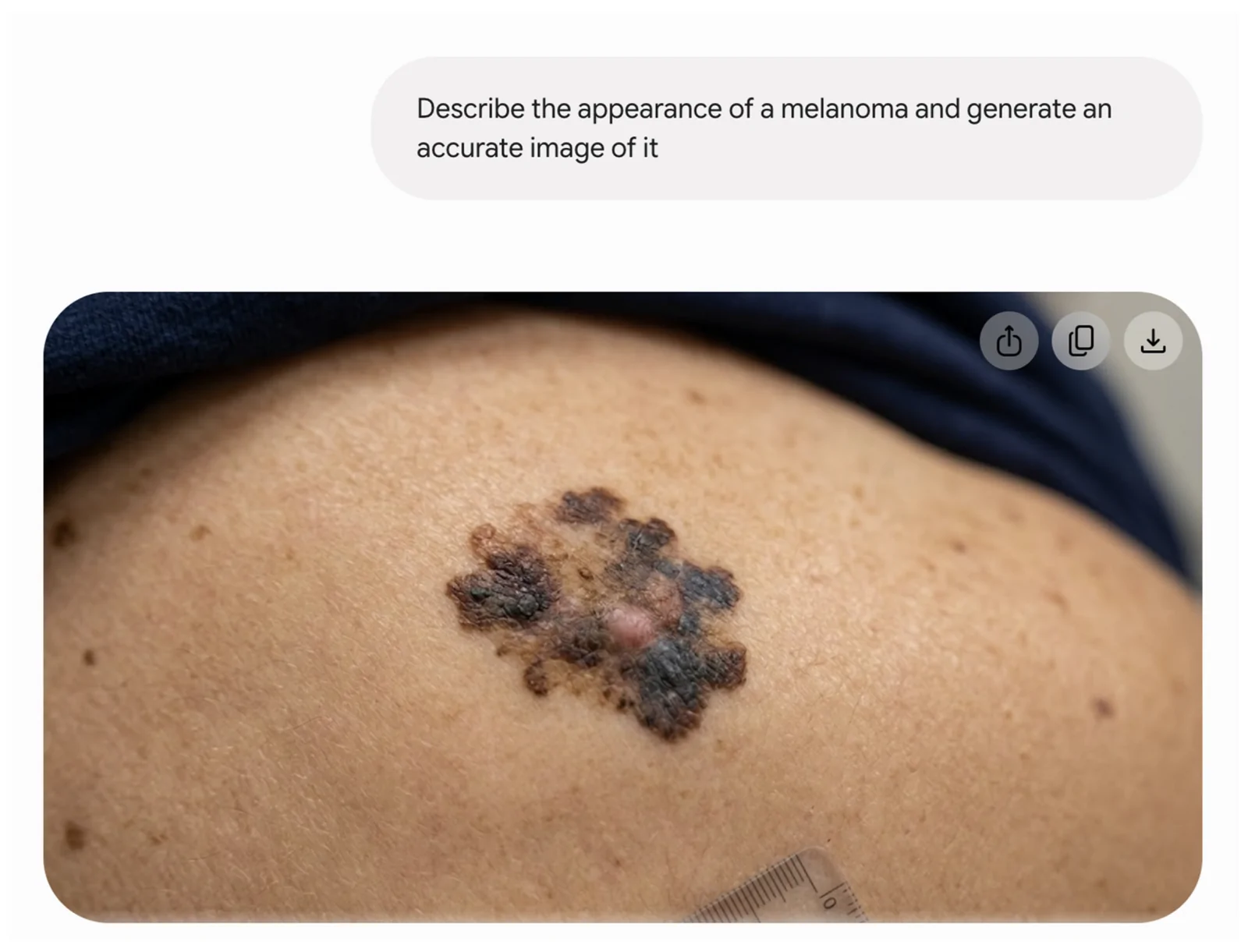
Gemini melanoma images generated.

**Table 1:** Prompts.

Prompt Number	Prompt
1	Describe the appearance of BCCs and generate an accurate image of it
2	Detail the gold-standard surgical treatment for BCCs
3	Describe the appearance of SCCs and generate an accurate image of it
4	Detail the gold-standard surgical treatment for SCCs
5	Describe the appearance of a melanoma and generate an accurate image of it
6	Detail the gold-standard surgical treatment for melanomas

**Table 2:** Likert scales for graphical responses for clinical representativeness and accuracy.

Prompt	Likert Criteria	ChatGPT	Gemini
1	Clinical Representativeness	4	3
	Accuracy	4	2
3	Clinical Representativeness	3	1
	Accuracy	3	2
5	Clinical Representativeness	4	3
	Accuracy	5	2

**Table 3:** Likert scales for textual responses for relevance and accuracy.

Prompt	Likert Criteria	ChatGPT	Gemini	Claude (Anthropic)	Copilot
Prompt 2	Relevance	5	4	4	4
	Accuracy	4	3	4	4
Prompt 4	Relevance	5	4	5	4
	Accuracy	3	4	4	4
Prompt 6	Relevance	5	4	5	5
	Accuracy	5	4	4	5

**Table 4:** Weighted Cohen’s kappa for Likert criteria scores of both the generated images and generated textual responses.

Domain	Rated Items	Exact Agreement, *n* (%)	Agreement Within 1 Point, *n* (%)	Weighted Cohen’s Kappa
Generated images				
Clinical representativeness	6	3 (50.0%)	6 (100%)	0.77 (0.33–0.89)
Accuracy	6	5 (83.3%)	6 (100%)	0.93 (0.40–1.00)
Textual responses				
Relevance	12	9 (75.0%)	12 (100%)	0.50 (0.00–1.00)
Accuracy	12	10 (83.3%)	12 (100%)	0.67 (0.00–1.00)

**Table 5:** Flesch Reading Ease Score (N/A represents a prompt that was requesting a graphical response).

Objective Linguistic Characteristics	ChatGPT	Gemini	Claude (Anthropic)	Copilot
Prompt 1	N/A	N/A	N/A	N/A
Prompt 2	14.3	30.5	31.7	39.0
Prompt 3	N/A	N/A	N/A	N/A
Prompt 4	11.9	19.5	18.5	36.8
Prompt 5	N/A	N/A	N/A	N/A
Prompt 6	21.3	46.5	37.9	47.7
Mean (SD)	15.83 (±4.88)	32.17 (±13.58)	29.37 (±9.91)	41.17 (±5.76)

**Table 6:** Flesch–Kincaid Grade Level (N/A represents a prompt that was requesting a graphical response).

Objective Linguistic Characteristics	ChatGPT	Gemini	Claude (Anthropic)	Copilot
Prompt 1	N/A	N/A	N/A	N/A
Prompt 2	12.8	12.8	12.4	9.6
Prompt 3	N/A	N/A	N/A	N/A
Prompt 4	13.8	12.9	13.3	10.4
Prompt 5	N/A	N/A	N/A	N/A
Prompt 6	12.2	8.7	10.0	8.3
Mean (SD)	12.93 (±0.81)	11.47 (±2.40)	11.90 (±1.71)	9.43 (±1.06)

**Table 7:** Coleman–Liau Index (N/A represents a prompt that was requesting a graphical response).

Objective Linguistic Characteristics	ChatGPT	Gemini	Claude (Anthropic)	Copilot
Prompt 1	N/A	N/A	N/A	N/A
Prompt 2	19.76	16.06	16.23	17.08
Prompt 3	N/A	N/A	N/A	N/A
Prompt 4	19.55	19.39	19.29	17.00
Prompt 5	N/A	N/A	N/A	N/A
Prompt 6	17.37	15.65	16.74	15.63
Mean (SD)	18.89 (±1.32)	17.03 (±2.05)	17.42 (±1.64)	16.57 (±0.82)

**Table 8:** DISCERN score.

DISCERN Score	ChatGPT	Gemini	Claude (Anthropic)	Copilot
Prompt 1	N/A	N/A	N/A	N/A
Prompt 2	35	33	38	32
Prompt 3	N/A	N/A	N/A	N/A
Prompt 4	41	36	43	33
Prompt 5	N/A	N/A	N/A	N/A
Prompt 6	45	38	50	43
Mean (SD)	40.33 (±5.03)	35.67 (±2.52)	43.67 (±6.03)	36.00 (±6.08)

## Data Availability

All data are available in this manuscript.

## Supplementary Materials

The following supporting information can be downloaded at https://www.mdpi.com/article/10.3390/jcm15218396/s1. Figure S1.1: ChatGPT BCC textual responses, Figure S1.2: ChatGPT SCC textual responses, Figure S1.3: ChatGPT melanoma textual responses, Figure S2.1: Gemini BCC textual responses, Figure S2.2: Gemini SCC textual responses, Figure S2.3: Gemini melanoma textual responses, Figure S3.1: Claude (Anthropic)’s BCC image generation response, Figure S3.2: Claude (Anthropic)’s generated BCC textual response, Figure S3.3: Claude (Anthropic)’s SCC image generation response, Figure S3.4: Claude (Anthropic)’s generated SCC textual response, Figure S3.5: Claude (Anthropic)’s melanoma image generation response, Figure S3.6: Claude (Anthropic)’s generated melanoma textual response, Figure S4.1: Copilot BCC image generation response, Figure S4.2: Copilot generated BCC textual response, Figure S4.3: Copilot SCC image generation response, Figure S4.4: Copilot generated SCC textual response, Figure S4.5: Copilot melanoma image generation response, Figure S4.6: Copilot generated melanoma textual response.

## Abbreviations

The following abbreviations are used in this manuscript:

AI	Artificial Intelligence
LLM	Large Language Model
CA	California
WA	Washington
USA	United States of America
cBCC	Cutaneous Basal Cell Carcinoma
cSCC	Cutaneous Squamous Cell Carcinoma
